# A minimal SufB_2_C_2_ complex functions as a [4Fe-4S] cluster scaffold in methanogenic archaea

**DOI:** 10.1128/spectrum.02134-25

**Published:** 2025-10-08

**Authors:** Cuiping Zhao, Nana Shao, Nicole Bryer, Haotian Chen, William B. Whitman, David J. Vinyard, Yuchen Liu

**Affiliations:** 1 Department of Biological Sciences, Louisiana State University 124526, Baton Rouge, Louisiana, USA; 2 Department of Microbiology, University of Georgia 189270, Athens, Georgia, USA; 3 Cain Department of Chemical Engineering, Louisiana State University 5779, Baton Rouge, Louisiana, USA; North Carolina State University, Raleigh, North Carolina, USA

**Keywords:** iron-sulfur protein, methanogen, SufB, SufC, MmpX

## Abstract

Iron-sulfur clusters are essential cofactors in all domains of life, yet their biogenesis in obligately anaerobic archaea remains poorly understood. Here, we characterized the minimal two-protein SUF system in methanogenic archaea, composed solely of SufB and SufC. Using *Methanococcus maripaludis* as a model, we demonstrate that the SUF proteins from its native host form a stable SufB_2_C_2_ heterotetramer that binds a [4Fe-4S] cluster via three conserved cysteines in SufC. Mutations of conserved cysteine and histidine residues of SufB do not impair cluster binding. The complex interacts with the SAM-containing methanogenesis marker protein 10 (MmpX), suggesting direct Fe-S cluster transfer from SufB_2_C_2_ to target proteins. Mutational analysis of *Methanothermococcus thermolithotrophicus* proteins confirmed that SufC is the primary cluster-binding component, while SufB enhances ATPase and cluster transfer activities. Evolutionary comparisons suggest that this two-protein SUF system represents an ancestral form of Fe-S cluster biogenesis.

**IMPORTANCE:**

Fe-S clusters are ancient and indispensable cofactors, yet their biosynthesis in obligately anaerobic archaea remains underexplored. This study provides mechanistic and evolutionary insights into the Fe-S cluster assembly machinery in methanogenic archaea. Unlike the bacterial six-component SUF systems, this minimal two-component SUF system (SufB_2_C_2_) operates without auxiliary proteins. Our findings expand the known diversity of Fe-S cluster biogenesis machineries and shed light on a potential evolutionary precursor adapted to the Earth’s ancient anoxic environments. It also provides a foundation for engineering minimal Fe-S cluster biosynthesis pathways in synthetic biology applications.

## INTRODUCTION

Iron-sulfur (Fe-S) clusters are among the most ancient and versatile inorganic cofactors, found in all domains of life. They enable redox chemistry and Lewis acid-type catalysis, supporting diverse biochemical processes in bioenergetics, biosynthesis, and genetic regulation (1, 2). The biosynthesis of Fe-S cluster-containing proteins involves two general steps: cluster assembly and transfer (3, 4). During assembly, a cysteine desulfurase—typically a pyridoxal-5′-phosphate-dependent enzyme—extracts sulfur from free l-cysteine (5), while the physiological iron donor remains uncertain (6). Iron and sulfur are then combined on a scaffold protein to form a nascent cluster *de novo* (7, 8). In the subsequent transfer step, the preassembled cluster is delivered to target apo-proteins either directly from the scaffold, often with assistance from ATP-dependent chaperones, or indirectly via Fe-S cluster carrier proteins (6).

The protein machineries responsible for Fe-S cluster biosynthesis vary across organisms and are influenced by environmental conditions. Bacteria have three known machineries: the NIF, ISC, and SUF systems (9, 10). The SUF system is particularly diverse among microorganisms. The *Escherichia coli* SUF operon encodes six proteins: SufA, SufB, SufC, SufD, SufS, and SufE (11, 12). The SufS-SufE complex functions as a cysteine desulfurase, donating sulfur to the scaffold complex. SufB and its homolog SufD each associate with one SufC ATPase to form a SufBC_2_D complex, which serves as the scaffold for [4Fe-4S] cluster assembly (13, 14). Specifically, SufB accepts sulfur from SufE only in the presence of SufC (15), while SufD is proposed to facilitate iron entry into the scaffold complex (16). The Fe-S cluster is thought to be coordinated at the SufB/D heterodimer interface by three conserved residues: SufB^C405^, SufB^E434^, and SufD^H360^ (17). SufC, a member of the ABC ATPase superfamily, contains the characteristic Walker A and B motifs along with an ATP-binding cassette (ABC signature motif) (18, 19). Structural studies suggest that a unique Q-loop on SufC mediates interactions with SufB or SufD (19). SufA is proposed to function as a cluster carrier, delivering Fe-S clusters to target apo-proteins (13, 20).

Methanogenic archaea are obligately anaerobic that rely extensively on Fe-S cluster proteins for methanogenesis and biosynthesis (21). For example, *Methanococcus maripaludis* contains ~15-fold more Fe-S clusters than aerobically grown *E. coli* (22). However, the canonical Fe-S cluster biosynthesis systems found in bacteria and eukaryotes are largely absent in archaea (21). Only three Fe-S cluster biogenesis proteins—Nbp35/ApbC/Mrp, SufB, and SufC—are conserved across nearly all archaea lineages (23, 24). In *M. maripaludis*, Nbp35/ApbC/Mrp binds a [4Fe-4S] cluster and can transfer it to apo-proteins (25). However, its deletion does not impair growth, suggesting it functions as a nonessential cluster transfer protein (25).

Recent studies have proposed that a SUF-like minimal system (SMS), composed of SufB and SufC, represents the ancestral SUF system present in the Last Universal Common Ancestors (LUCAs) (26–29). It is retained in most methanogenic archaea and is likely the sole Fe-S cluster biogenesis machinery in *Methanococcales* and *Methanopyrales* (26). Transposon mutagenesis data indicate that *sufB* (locus tag: MMP1169) and *sufC* (locus tag: MMP1168) are likely to be essential in *M. maripaludis* (30). However, deletion of the *sufCB* gene clusters in *Methanosarcina acetivorans*, which also has the ISC system (31), showed no growth defect (32). This study raised questions about the *in vivo* function of SufB and SufC in methanogens. Here, we present comprehensive biochemical and spectroscopic characterizations of SufB and SufC proteins from their native host *M. maripaludis*, demonstrating that the SufB_2_C_2_ complex is a functional [4Fe-4S] cluster scaffold. This methanococcal minimal SUF machinery may represent a living model of early anaerobic life, adapted to environments rich in ferrous iron and reduced sulfur (21, 23).

## MATERIALS AND METHODS

### Strains and culture conditions

All strains used in this study are listed in Table S3. *M. maripaludis* was cultured in 28 mL aluminum-sealed tubes containing 5 mL McC medium under 275 kPa of H₂:CO₂ (4:1, vol/vol) at 37°C, as previously described (33). For larger-scale cultures, 100 mL volumes were grown in 1 L bottles pressurized to 138 kPa with H₂:CO₂ (4:1, vol/v). Iron was supplied at a final concentration of 30 µM by adding Fe(NH_4_)_2_(SO_4_)_2_ from a 5 mM stock solution. Puromycin (2.5 µg/mL) was added when required. Prior to inoculation, sodium sulfide was added as the sulfur source at a final concentration of 3 mM from a 100 mM stock solution.

*E. coli* strains were grown aerobically at 37°C in Luria-Bertani (LB) medium. Antibiotics were used at the following concentrations when necessary: ampicillin (100 µg/mL), kanamycin (25 µg/mL), spectinomycin (100 µg/mL), and chloramphenicol (100 µg/mL).

### Production and anoxic purification of MMP SufB-SufC and MmpX in *M. maripaludis*

The *M. maripaludis sufCB* operon (SufC: UniProtKB Q6LY25; SufB: UniProtKB Q6LY24) was cloned into the pMEV4mTs vector with an N-terminal Strep-tag. The *mmpX* gene (UniProtKB Q6L × 00) was cloned into the Pst-pMEV4mTs vector with a C-terminal TAP-tag (3 × FLAG and Twin-Strep). Expression vectors were transformed into the *M. maripaludis* strain S0001 and a Δ*mmpX* deletion strain using PEG-mediated transformation (34). Protein purification was performed as previously described (35).

### Production and anoxic purification of MTH SufB and SufC in *E. coli*

*M. thermolithotrophicus sufC* (locus tag: F555DRAFT_01626) and *sufB* (F555DRAFT_01627) were cloned into pET15b (N-terminal His₆-tag) and pDCH (C-terminal His₆-tag), respectively. Co-expression of His₆-SufC and untagged SufB was achieved by co-transforming both plasmids into *E. coli* Rosetta 2(DE3) strain (Novagen). Mutants were generated using the QuikChange mutagenesis kit (Agilent).

Cultures were grown in LB supplemented with 200 µM ammonium iron(III) citrate and 30 µM L-methionine at 37°C with 250 rpm shaking until OD₆₀₀ reached 0.6–0.8. Protein expression was induced with 0.1 mM isopropyl β-d-1-thiogalactopyranoside (IPTG) and continued overnight at 25°C with 110 rpm rotation speed. All purification steps were performed anoxically in a Coy chamber with an atmosphere of 95% (vol/vol) N_2_ and 5% (vol/vol) H_2_. Cells were harvested by centrifugation (4,000 × *g*, 10 min, 4°C), resuspended in binding buffer (50 mM sodium HEPES, 300 mM NaCl, 5 mM MgCl₂, 20 mM imidazole, pH 7.5), and lysed by sonication. Lysates were applied to Ni-NTA columns (Qiagen), washed, and eluted with 200 mM imidazole. Purified proteins were supplemented with 20% (vol/vol) glycerol and stored at –80°C.

### Protein identification by mass spectrometry

Purified proteins were separated by SDS-PAGE and visualized with silver staining. Protein bands of interest were excised, destained, and subjected to in-gel digestion with trypsin. A stock trypsin solution was prepared by dissolving 20–25 mg of trypsin in 200 µL of 1 mM HCl, yielding a final concentration of 100–125 ng/μL. This stock was then diluted 1:10 with 25 mM ammonium bicarbonate containing 1% acetonitrile. Dried gel bands were rehydrated with ~10 µL of the working trypsin solution and incubated at room temperature for 20 min to allow enzyme infusion. Excess trypsin was removed by two washes with 25 mM ammonium bicarbonate, followed by the addition of 10–20 μL of 25 mM ammonium bicarbonate to maintain hydration during digestion. Digestion was carried out at 37°C for 4–6 hours and terminated by adding 25 µL of 10% formic acid. The supernatant was collected, and additional peptides were recovered by two sequential extractions with 25 µL of 50% (vol/vol) acetonitrile in 50 mM ammonium bicarbonate, followed by a final extraction with 25–50 μL of 100% acetonitrile. All extracts were pooled and concentrated to ~5 µL using a vacuum centrifuge.

The liquid chromatography-mass spectrometry (LC-MS) analysis was performed at the Louisiana State University Mass Spectrometry Facility using an amaZon speed ETD ion trap mass spectrometer (Bruker Daltonics, Billerica, MA, USA). Sample solutions were prepared by adding 10 µL of a 50:50 (vol/vol) mixture of acetonitrile and 0.1% formic acid in water to the dried peptide samples. The sample solution of 1 µL was loaded onto a C18 PepMap column (75 µm ID, 15 cm, LC Packings). Peptides were separated at a flow rate of 300 nL/min using a 40 minute gradient of solvent B (95% acetonitrile, 5% water, 0.1% formic acid): solvent B was increased from 0% to 5% in 30 seconds, ramped from 5% to 55% in 22 min, increased to 95% in 4 min, held at 95% for 2.5 min, returned to 5% in 1 min, and equilibrated at 5% for 10 min before the next injection. The LC system interfaced directly with the ion trap mass spectrometer via a nanoESI spray source. MS detection was performed in enhanced resolution full-scan mode under positive ionization at a scan speed of 8,100 m/z/s. Data processing and deconvolution were performed using the Compass DataAnalysis software (Bruker Daltonics). Proteins were identified using the MASCOT search engine (Matrix Science, London, UK) against the NCBIprot database, allowing a mass deviation of <0.2 Da and one missed cleavage per peptide.

### Analytical and spectroscopic measurements

All measurements were performed in triplicate. Protein concentrations were determined using the BCA Protein Assay Kit (Pierce). Iron content was measured using the Quantichrom Iron Assay Kit (BioAssay Systems). UV-visible spectra were recorded on a Nanodrop 2000c spectrophotometer under anoxic conditions using sealed cuvettes (1 cm path length).

X-band EPR spectra were recorded at 7–10 K on a Bruker EMX spectrometer equipped with a standard resonator and Oxford ESR-900 helium flow cryostat. Multiple microwave powers were tested so that resonances were measured under nonsaturating conditions. The *g* values were determined by simulating spectra using EasySpin 5.2.20 (36).

### Size-exclusion chromatography

Size-exclusion chromatography (SEC) was performed using a Superdex 200 10/300 Gl column (GE Healthcare) equilibrated with 50 mM sodium HEPES, 200 mM NaCl, and 5 mM MgCl₂ (pH 7.5). The column was calibrated with molecular weight standards (29–700 kDa, Sigma-Aldrich). Protein samples were pretreated with 10 mM EDTA and 10 mM DTH overnight, centrifuged (14,000 × *g*, 10 min) to remove the precipitate, and loaded onto the column. Fractions were analyzed by SDS-PAGE.

### ATPase assay

ATPase activity was measured using malachite green reagent to detect released Pi. Reactions were performed for 5 min with 0.5 µM MTH SufB₂C₂ or 20 µM MTH SufC in buffer (50 mM sodium HEPES, 300 mM NaCl, 5 mM MgCl₂, pH 7.5) at RT, 37°C, or 60°C with 0-2 mM ATP. Reactions were stopped with 50 mM EDTA. Apo-proteins were prepared by overnight incubation with 10 mM DTH and 10 mM EDTA at 4°C, followed by buffer exchange using a PD MiniTrap G-25 column (GE Healthcare) pre-equilibrated with the buffer containing 50 mM sodium HEPES (pH 7.5), 300 mM NaCl, and 5 mM MgCl_2_. Kinetic parameters (V_max_, K_m_) were determined with 0–2 mM ATP and plotted using KaleidaGraph.

### Fe-S cluster transfer assay

All steps were performed anoxically in a Coy chamber with an atmosphere of 95% (vol/vol) N_2_ and 5% (vol/vol) H_2_. His-tagged *E. coli* aconitase B (AcnB) was purified from strain JW0114 from the ASKA collection (37) following the purification procedure as described (38). Apo-AcnB was prepared by treatment with 10 mM EDTA and 10 mM DTH overnight at 4°C, followed by buffer exchange using a PD MiniTrap G-25 column (GE Healthcare) pre-equilibrated with the buffer containing 50 mM sodium HEPES (pH 7.5), 300 mM NaCl, and 5 mM MgCl_2_.

For cluster transfer, apo-AcnB (0.375 nmol) and Strep-tagged MMP SufB₂C₂ (0.75 nmol [4Fe-4S]) were pretreated with 5 mM 1, 4-dithiothreitol (DTT) for 30 min, mixed in 35 µL total volume, and incubated for 20 min at RT.

### Aconitase activity assay

Aconitase activity was measured by adding 10 µL of the reaction to a 100 µL assay mixture containing 50 mM sodium HEPES (pH 7.5), 300 mM NaCl, 5 mM MgCl₂, 0.25 mM NADP^+^, 25 mM sodium citrate, 0.3 mM MnCl₂, and 1 µM His-tagged *E. coli* isocitrate dehydrogenase (from strain JW1122 from the ASKA collection). NADPH formation was monitored at 340 nm.

### Chemical reconstitution of Fe-S clusters

All steps were performed anoxically in a Coy chamber with an atmosphere of 95% (vol/vol) N_2_ and 5% (vol/vol) H_2_. Proteins were incubated with 10 mM DTH and 10 mM EDTA overnight at 4°C to remove native clusters, followed by buffer exchange using a PD MiniTrap G-25 column (GE Healthcare) pre-equilibrated with the buffer containing 50 mM sodium HEPES (pH 7.5), 300 mM NaCl, and 5 mM MgCl_2_. Reconstitution was performed by incubating the protein with 5 mM DTT for 1 h at RT, followed by dropwise addition of ferrous ammonium sulfate and sodium sulfide (8-fold molar excess of the protein concentration). After 2 h incubation, excess reagents were removed using a PD MiniTrap G-25 column.

## RESULTS

### SufB and SufC from methanogens form a SufB_2_C_2_ complex *in vivo*

We investigated the SufB and SufC proteins from two methanogenic archaea: *Methanococcus maripaludis* (MMP), a widely used model organism, and *Methanothermococcus thermolithotrophicus* (MTH). Protein sequence alignments revealed that both the SufB and SufC proteins from these species share 74% identity. Both MMP SufB and MTH SufB possess 26% identity with *E. coli* SufB and 30% identity with *E. coli* SufD. The MMP SufC shares 39% identity with *E. coli* SufC, while the MTH SufC shows 38% identity with *E. coli* SufC.

**Fig 1:**
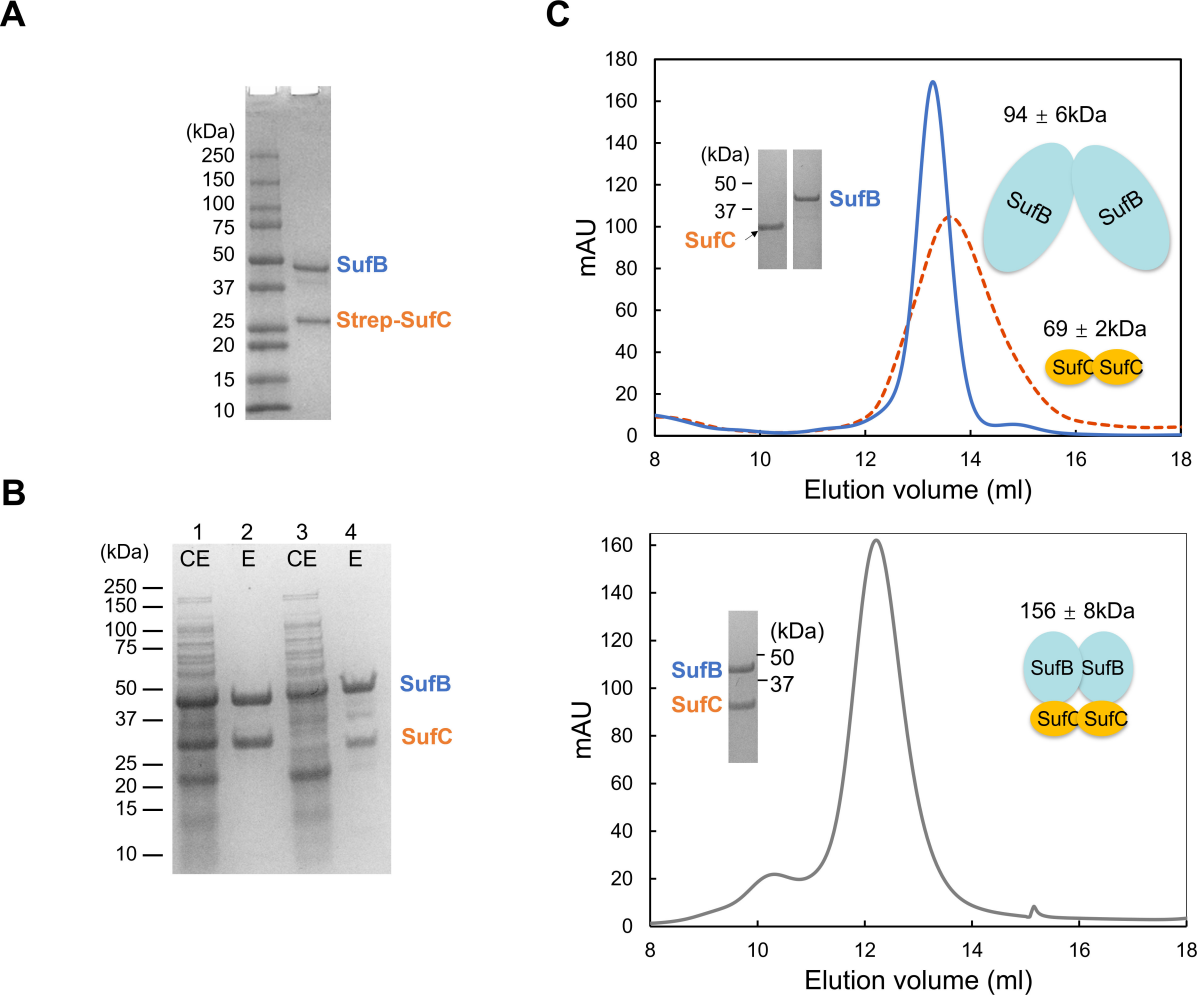
SufB and SufC form a SufB_2_C_2_ complex in methanogens. (**A**) Pull-down assay in *M. maripaludis* showing interaction between MMP SufB and SufC. The *sufCB* operon was expressed with an N-terminal Strep-tag on SufC. Following anoxic purification, SDS-PAGE analysis revealed co-purification of SufB with SufC. (**B**) Pull-down assay in *E. coli* showing interaction between MTH SufB and SufC. Lane 1 and Lane 3 represent crude extracts (CE) corresponding to Lane 2 and Lane 4, respectively. Lane 2: elution (E) from Ni-NTA purification of His_6_-tagged MTH SufC co-expressed with untagged MTH SufB. Lane 4: elution (E) from Ni-NTA purification of His_6_-tagged MTH SufB co-expressed with untagged MTH SufC. All gels were stained with Coomassie blue. (**C**) SEC analysis of MTH SufB, SufC, and the SufBC complex. *Top panel*: SEC profiles of purified MTH SufB (blue solid line) and SufC (red dashed line). Embedded SDS-PAGE gel shows bands corresponding to SufC (left) and SufB (right). Cartoon illustrations depict SufB and SufC dimers. *Bottom panel*: SEC profile of the co-expressed MTH SufBC complex (gray line). Embedded SDS-PAGE gel shows the bands for SufC (lower) and SufB (upper). The cartoon illustrates the SufB_2_C_2_ complex. Data are mean ± SDs (*n* = 3).

To study complex formation, we expressed the MMP *sufCB* operon in *M. maripaludis* and also heterologous in *E. coli*. While the MMP SufB and SufC proteins were insoluble in *E. coli*, the MTH homologs were successfully expressed as soluble proteins. Three lines of evidence support the formation of a SufB/C complex in methanogens. (i) Native pull-down in *M. maripaludis:* the MMP *sufCB* operon with an N-terminal Strep-tag on SufC was cloned and expressed. Anoxic purification followed by SDS-PAGE analysis revealed that the MMP SufB co-purified with SufC (Fig. 1A). Protein identities were confirmed by mass spectrometry. (ii) Heterologous pull-down in *E. coli*: in one experiment, the His_6_-tagged MTH SufC and untagged MTH SufB were co-expressed from separate vectors; in another, His_6_-tagged MTH SufB was co-expressed with untagged MTH SufC. Pull-down assays in both cases confirmed that *in vivo* interactions between SufB and SufC (Fig. 1B; Fig. S1). (iii) SEC: the purified MTH SufB eluted as a major peak at ~94 ± 6 kDa, consistent with a SufB dimer (calculated monomeric MW = 46.5 kDa). MTH SufC eluted at ~69 ± 2 kDa, consistent with a SufC dimer (calculated monomeric MW = 30 kDa). The purified MTH SufB/C complex eluted at ~156 ± 8 kDa, consistent with a SufB_2_C_2_ heterotetramer. These results collectively demonstrate that SufB and SufC form a stable SufB_2_C_2_ complex in methanogens.

### The SufB_2_C_2_ complex binds a [4Fe-4S] cluster in *M. maripaludis*

**Fig 2:**
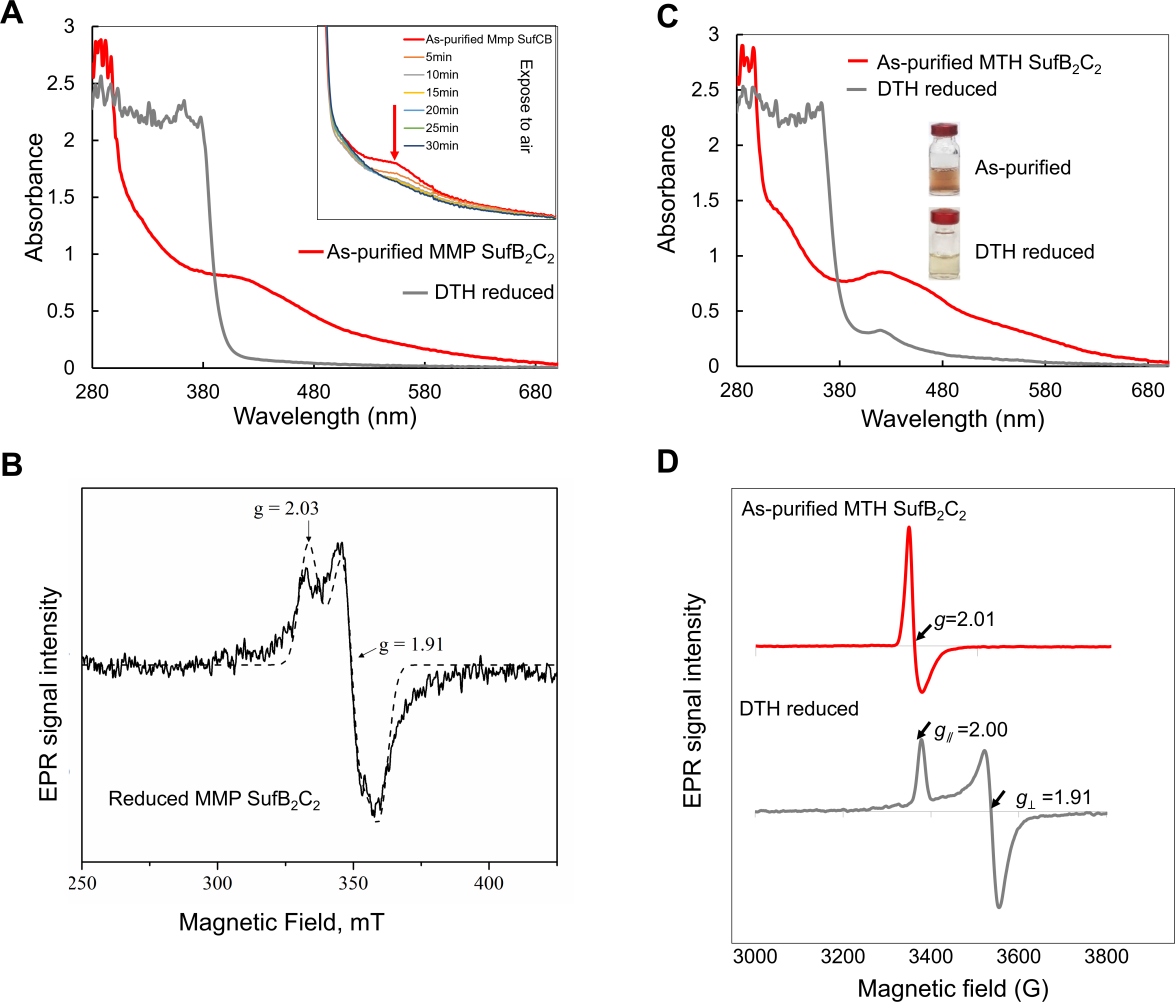
The MMP SufB_2_C_2_ complex binds a [4Fe-4S] cluster. (**A**) UV-visible spectra of anoxically purified MMP SufB_2_C_2_ complex in the as-purified (red) and DTH-reduced (gray) states. (*Inset*) Spectra of the protein after exposure to air. The red arrow indicates the decrease in absorbance at ~420 nm upon oxidation. (**B**) X-band EPR spectrum of the anoxically purified MMP SufB_2_C_2_ complex after DTH reduction. The spectrum was simulated as a nearly axial species with *g_∥_* =2.03 and *g*_⊥_ =1.91 (dashed trace). Experimental conditions: microwave power, 1 mW; microwave frequency, 9.474 GHz; modulation amplitude, 10 G; temperature, 10 K. (**C**) UV-visible spectra of anoxically purified MTH SufB_2_C_2_ complex in the as-purified (red) and DTH-reduced (gray) states. (*Inset*) Photographs of protein solutions before and after DTH reduction. (**D**) X-band EPR spectra of the MTH SufB_2_C_2_ complex in the as-purified (red) and DTH-reduced (gray) states. Experimental conditions: microwave power, 1 mW; microwave frequency, 9.473 GHz; modulation amplitude, 10 G; temperature, 10 K.

Four lines of evidence support the presence of a Fe-S cluster in the methanogen SufB_2_C_2_ complex. (i) UV-visible spectroscopy: the anoxically purified MMP SufB_2_C_2_ complex exhibited a brownish color and a characteristic broad absorption peak at ~420 nm (Fig. 2A). Addition of 5 mM sodium dithionite (DTH) partially bleached the color and reduced the absorption (Fig. 2A). The cluster was also found to be labile upon air exposure (Fig. 2A). (ii) Iron quantification: chemical analysis revealed 1.6 ± 0.3 Fe atoms per protomer in the as-purified MMP SufB_2_C_2_ complex. (iii) Electron paramagnetic resonance (EPR) spectroscopy: the as-purified MMP SufB_2_C_2_ displayed a minor signal centered at *g* = 2.003, attributed to an organic radical contaminant. Upon reduction with 5 mM sodium DTH, the complex exhibited a rhombic EPR signal with *g_∥_* ~2.03 and *g*_⊥_ ~1.91, characteristic of a [4Fe-4S]^1+^ cluster (*S*_tol_ = ½) (Fig. 2B). The organic radical signal is overlaid with the [4Fe-4S] cluster signal. (iv) Comparison with heterologously expressed MTH SufB_2_C_2_: the MTH SufB_2_C_2_ complex, anoxically purified from *E. coli*, showed similar properties to MMP SufB_2_C_2_. It had a brownish color, contained 1.54 ± 0.32 Fe per protomer, and displayed a broad UV-vis absorption peak at ~420 nm (Fig. 2C). DTH treatment similarly reduced the absorbance. EPR analysis of the as-purified MTH SufB_2_C_2_ displayed a signal at *g* ~ 2.01, consistent with a cubic [3Fe-4S]^1+^ cluster (*S*_tol_ = ½), whereas the proteins reduced with 5 mM sodium DTH showed a signal with *g_∥_* ~2.00 and *g*_⊥_ ~1.91, characteristic of a [4Fe-4S]^1+^ cluster (*S*_tol_ = ½) (Fig. 2D). Given that the MMP SufB_2_C_2_ complex purified from its native host contains only a [4Fe-4S] cluster, the [3Fe-4S] cluster observed in MTH SufB_2_C_2_ may result from partial degradation of a [4Fe-4S] cluster during purification.

### The MMP SufB_2_C_2_ complex can transfer a [4Fe-4S] cluster to apo-aconitase

**Fig 3:**
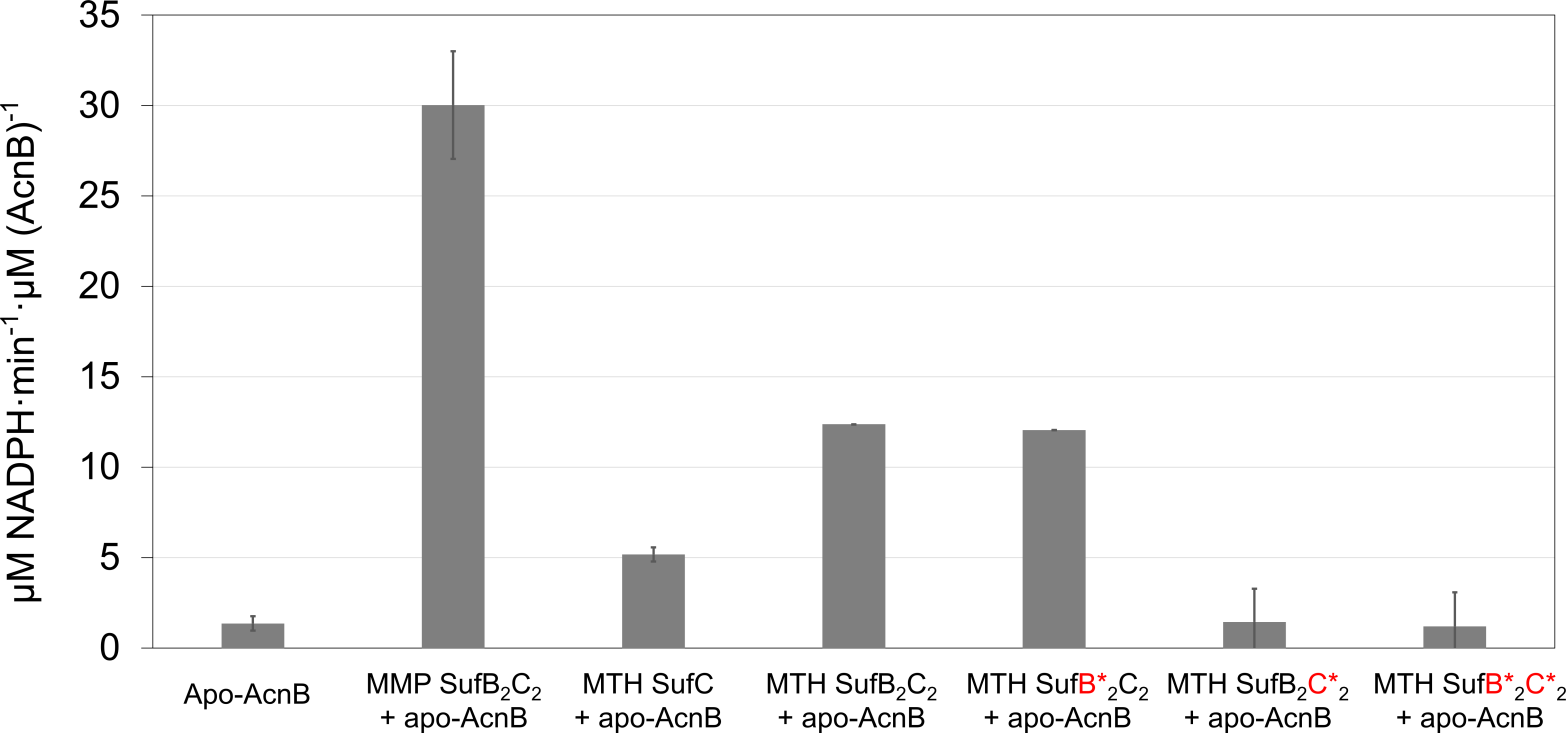
SufB_2_C_2_-bound [4Fe-4S] cluster activates apo-aconitase B (AcnB). The MMP SufB_2_C_2_ complex was expressed in *M. maripaludis*, while MTH SufC, MTH SufB_2_C_2_, and its mutants were expressed and purified from *E. coli*. The MTH SufB*_2_C_2_ complex carries the four SufB mutations (C145/175/318S, H346S), and the MTH SufB_2_C*_2_ complex carries three SufC mutations (C218/237/240S). The SufB*_2_C*_2_ complex carries both the SufB (C145/175/318S, H346S) and SufC (C218/237/240S) mutation. Fe-S cluster transfer assays were performed by incubating apo-AcnB with the as-purified SufB_2_C_2_ complexes, followed by activity measurement as described in the Materials and Methods section. Data are mean ± SDs (*n* = 3).

To assess the functional role of MMP SufB_2_C_2_ in Fe-S cluster transfer, we tested its ability to reconstitute the [4Fe-4S] cluster-dependent enzyme *E. coli* aconitase B (AcnB). Cluster transfer was monitored by measuring the restoration of AcnB catalytic activity. As expected, neither the as-purified MMP SufB_2_C_2_ nor apo-AcnB alone had detectable activity. However, co-incubation restored AcnB activity (Fig. 3), indicating successful [4Fe-4S] cluster transfer from MMP SufB_2_C_2_ to apo-AcnB. These results suggest that SufB_2_C_2_ functions as a scaffold capable of assembling and delivering Fe-S clusters to target proteins in methanogens.

### The SufB_2_C_2_ complex interacts with the Fe-S cluster protein MmpX in *M. maripaludis*

**Fig 4:**
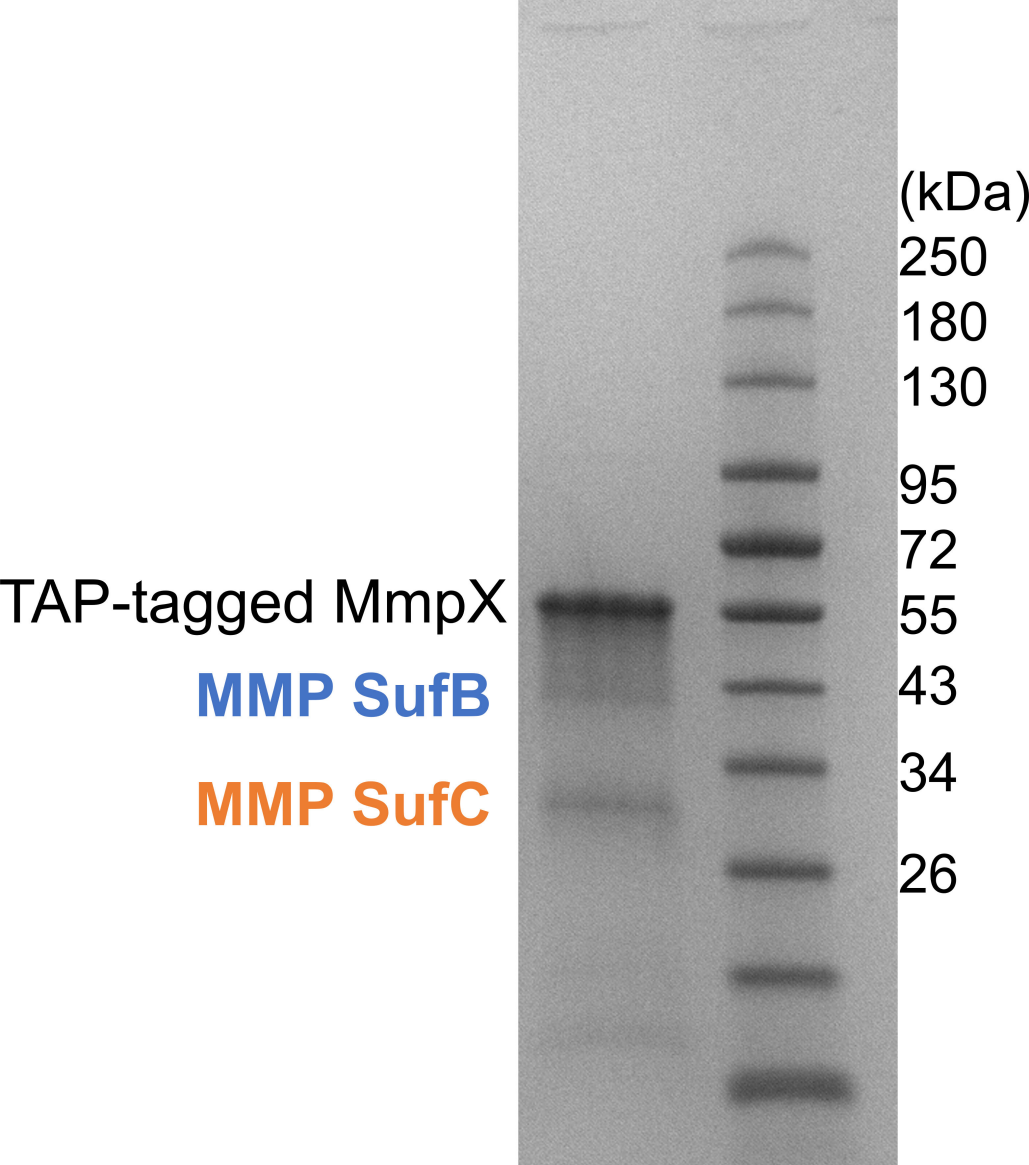
SufB and SufC co-purify with TAP-tagged MmpX in *M. maripaludis*. SDS-PAGE analysis of the purified TAP-tagged MmpX complex reveals co-purification of native MMP SufB and SufC. The gel was stained with Coomassie blue, and the protein identities were confirmed by mass spectrometry.

To investigate potential interactions between SufB_2_C_2_ and other Fe-S cluster proteins, we purified the FLAG_3_-Strep_2_-tagged methanogenesis marker protein 10 (MmpX) from its native host *M. maripaludis*. MmpX is a Fe-S cluster-containing radical SAM enzyme responsible for the posttranslational methylation of Arg in methyl coenzyme M reductase (39, 40). SDS-PAGE analysis revealed co-purification of SufB and SufC with MmpX (Fig. 4), suggesting a physical interaction. This implies that MMP SufB_2_C_2_ complex may serve as a scaffold for delivering [4Fe-4S] clusters to MmpX, facilitating its maturation *in vivo*.

### The [4Fe-4S] cluster is coordinated on three conserved cysteines of SufC

**Fig 5:**
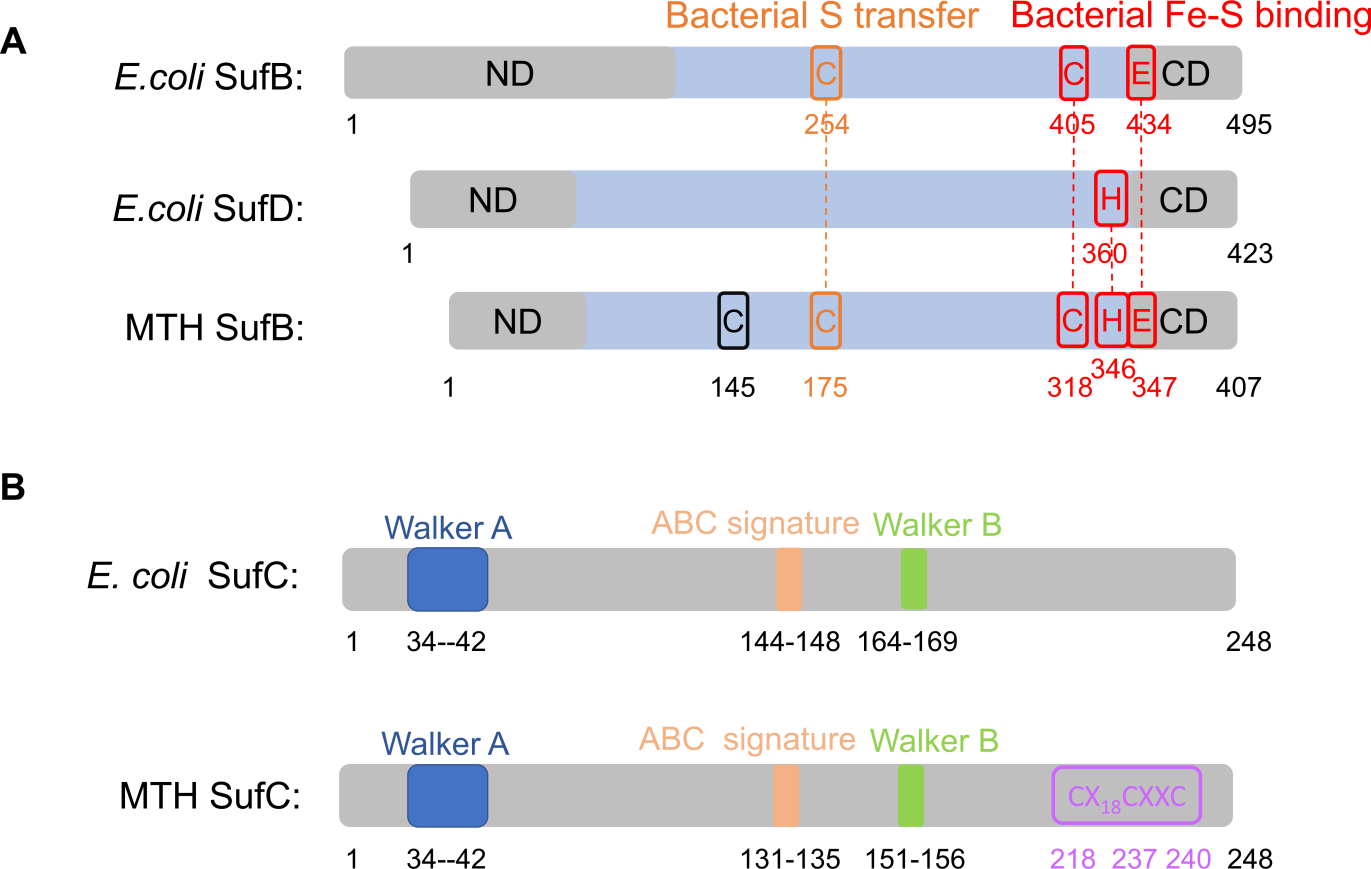
Sequence comparisons of *E. coli* and MTH SufB/C proteins. (**A**) Comparisons of *E. coli* SufB (NCBI Reference Sequence: WP_042190429.1) and SufD (NCBI Reference Sequence: WP_040083833.1) with MTH SufB (locus tag: F555DRAFT_01627) based on multiple sequence alignment. Residues predicted to participate in Fe-S cluster binding are highlighted in red, and those involved in bacterial sulfur transfer are labeled in orange. ND, N-terminal domain; CD, C-terminal domain. Amino acid position numbers are indicated below each scheme. (**B**) A comparison of *E. coli* SufC (locus tag: Ga0063390_04487) with MTH SufC (locus tag: F555DRAFT_01626) based on sequence alignment. Conserved Cys residues in archaeal SufC are highlighted in purple. Functional motifs Walker A (blue), Walker B (green), and ABC (orange) are labeled.

To determine the amino acid residues responsible for Fe-S cluster coordination, we performed site-directed mutagenesis of conserved residues in MTH SufB and SufC. In the *E. coli* SUF system, residues SufB^C405^, SufB^E434^, and SufD^H360^ are implicated in Fe-S cluster binding, and SufB^C254^ is likely involved in S transfer (17, 41). These four residues are highly conserved in archaeal homologs (Table S1). The corresponding residues in MTH SufB—C175, C318, H346, and E347—are labeled in Fig. 5A. Additionally, a conserved cysteine residue (C145) is present in methanogen SufB homologs (Fig. 5A; Fig. S2; Table S1). Notably, methanogen SufC homologs contain three highly conserved C-terminal cysteines (C218, C237, and C240) in a CX_n_CXXC motif (Fig. 5B; Fig. S2; Table S2). This motif is only present in some bacterial and non-methanogen archaeal SufC homologs (Table S2).

**Fig 6:**
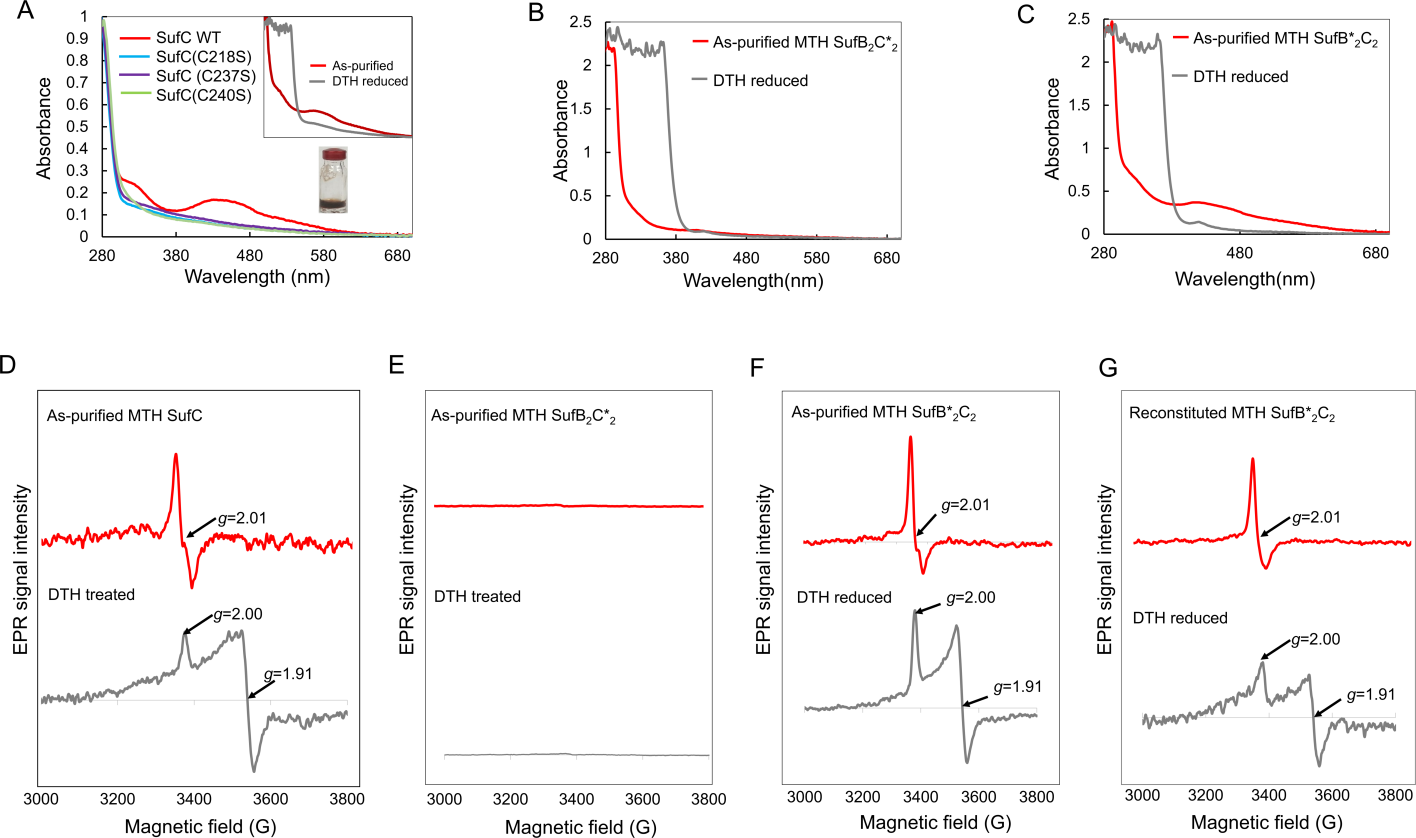
Three Cys residues in SufC coordinate the [4Fe-4S] cluster. (**A–C**) UV-visible spectra of anoxically purified proteins at 40 µM. (**A**) MTH SufC and its Cys→Ser variants (C218S, C237S, and C240S). The photograph shows the protein solution of as-purified SufC. (*Inset*) UV-visible spectra of MTH SufC in as-purified (red) and after DTH reduced (gray) states. (**B**) MTH SufB_2_C*_2_ complex carrying the triple SufC mutation (C218/237/240S). (**C**) MTH SufB*_2_C_2_ complex carrying four SufB mutations (C145/175/318S, H346S). (**D–G**) X-band EPR spectra of anoxically purified proteins in the as-purified (red) and the DTH reduced (gray) states. Experimental conditions: microwave power, 1 mW; microwave frequency, 9.474 GHz; modulation amplitude, 10 G; temperature, 10 K. (**D**) MTH SufC in the as-purified state shows a signal at *g* ~ 2.01, characteristic of a cubic [3Fe-4S]^1+^ cluster (*S*_tol_ = ½). Upon reduction with 5 mM sodium DTH, a rhombic signal appears with *g_∥_* ~2.00 and *g*_⊥_ ~1.91, consistent with a [4Fe-4S]^1+^ cluster (*S_tol_* = ½). (**E**) No EPR signal is detected in the MTHSufB_2_C*_2_ complex, indicating loss of cluster binding. (**F**) The MTH SufB*_2_C_2_ complex displays EPR signals similar to the wild-type complex. (**G**) The reconstituted MTH SufB*_2_C_2_ complex upon DTH reduction shows EPR features consistent with a [4Fe-4S]^1+^ cluster.

To assess their roles in Fe-S cluster binding, we individually mutated SufB^C145^, SufB^C175^, SufB^C318^, SufB^H346^, SufC^C218^, SufC^C237^, and SufC^C240^ to Ser. The mutant proteins were purified under anoxic conditions and characterized by UV-visible and EPR spectroscopy. Three lines of evidence support that the Fe-S cluster binds to SufC. (i) SufC alone binds the cluster, while SufB does not. As-purified MTH SufC exhibited a brownish color and contained 1.67 ± 0.14 iron per monomer. It displayed UV-visible (Fig. 6A) and EPR spectra (Fig. 6D) similar to the MTH SufB_2_C_2_ complex. (ii) SufC mutations abolished Fe-S cluster binding and transfer. The C218S, C237S, and C240S variants of MTH SufC lost their brownish color and UV-visible absorbance (Fig. 6A). Iron content in these mutants was below the detection limit (<0.024 Fe per mol protein). Furthermore, the MTHSufB_2_C*_2_ complex carrying the triple SufC mutation (C218/237/240S) lacked UV-visible (Fig. 6B) and EPR (Fig. 6E) signals and contained no detectable iron. This mutant complex also failed to activate apo-AcnB, indicating loss of cluster transfer capability (Fig. 3). (iii) SufB mutations do not impair Fe-S cluster binding and transfer. The MTH SufB*_2_C_2_ complex carrying four SufB mutations (C145/175/318S, H346S) retained UV-visible (Fig. 6C) and EPR (Fig. 6F) signals and activated apo-AcnB at levels comparable to the wild-type complex (Fig. 3). Furthermore, chemical reconstitution of EDTA-treated apo-MTH SufB*_2_C_2_ with an eight-fold molar excess of Fe^2+^ and S^2-^ successfully restored a [4Fe-4S], as evidenced by EPR spectra similar to the as-purified complex (Fig. 6G). This result confirms that the conserved Cys residues in SufB are non-essential for *de novo* cluster assembly.

Collectively, these results demonstrate that the labile and transferable [4Fe-4S] cluster in the MTH SufB_2_C_2_ complex is coordinated by the three conserved Cys residues in SufC—C218, C237, and C240, while the conserved residues in SufB are dispensable for cluster binding and transfer.

### SufB enhances the ATPase and Fe-S cluster transfer activities of SufC

**Fig 7:**
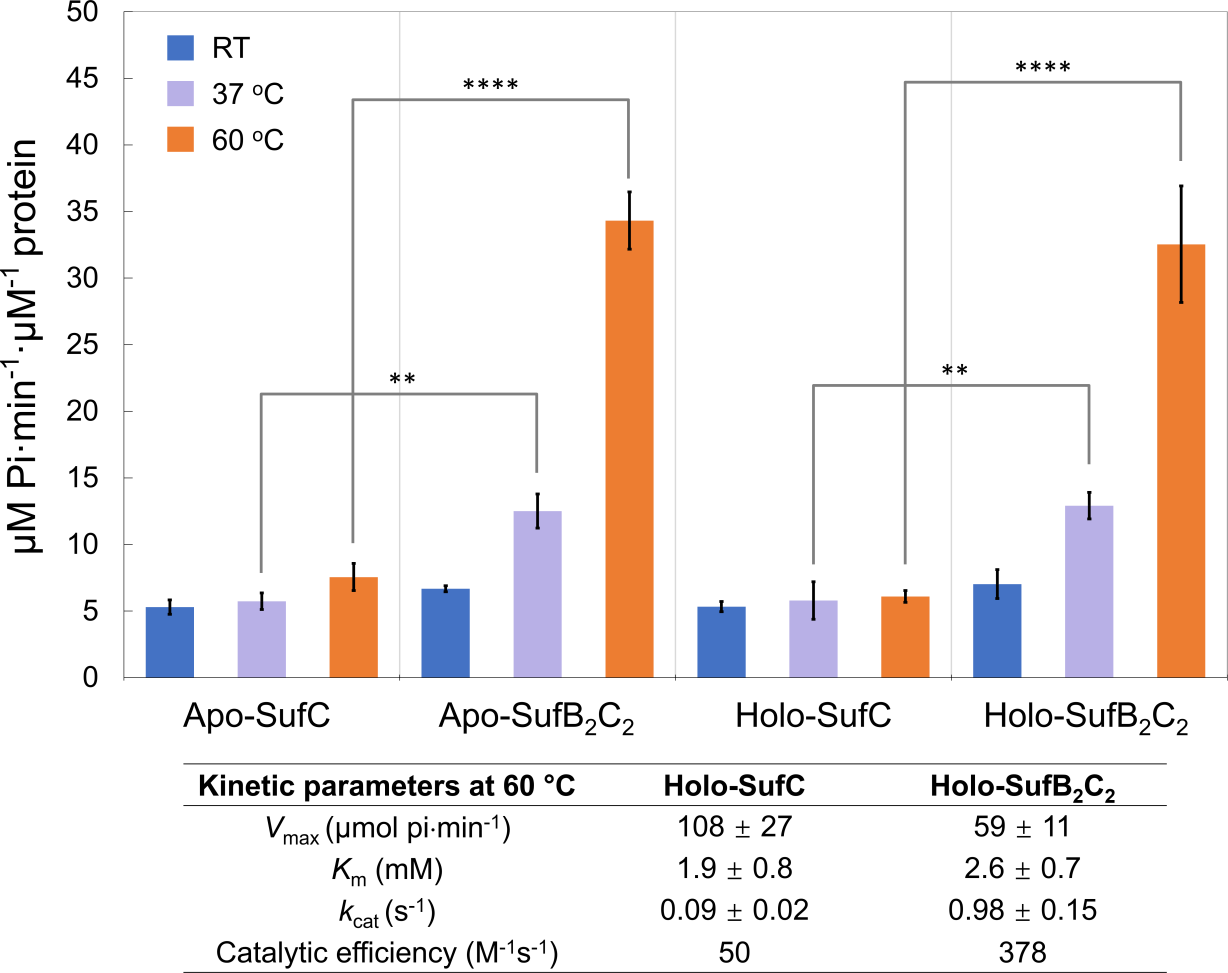
Temperature-dependent ATPase activity of MTH SufC and SufB_2_C_2_. ATPase activity was measured for 1 µM MTH SufC and 0.5 µM MTH SufB_2_C_2_ at room temperature (RT), 37°C, and 60°C under anoxic conditions in the presence of 2 mM ATP and 5 mM MgCl_2_. Apo-form proteins were prepared by overnight incubation with 10 mM DTH and 10 mM EDTA at 4°C, followed by buffer exchange using a G-25 column. The table below the figure summarizes kinetic parameters (*V*_max_, *K*_m_, *k*_cat_, and catalytic efficiency) for holo-forms of MTH SufC and SufB_2_C_2_ at 60°C. Kinetics were determined with 0-2 mM ATP and plotted with KaleidaGraph. Data are mean ± SDs (*n* = 3). Statistical significance: **P < 0.01, ****P < 0.0001.

SufC homologs in archaea contain three conserved motifs of ABC ATPases: Walker A and B and the ABC signature motif (Fig. 5B; Table S2) (19). To determine whether MTH SufC functions as an ATPase, we measured its activity using the malachite green assay. Given that *M. thermolithotrophicus* is a thermophile, assays were performed at 60°C in addition to room temperature (RT) and 37°C. Our results (Fig. 7) reveal the following. (i) MTH SufC alone exhibits ATPase activity. The purified MTH SufC possessed a specific activity of ~6 µM inorganic phosphate (Pi) formed⋅min^−1^⋅(μM SufC_2_)^−1^ at pH 7.5 in the presence of 5 mM MgCl_2_ across all tested temperatures. (ii) Complex formation with SufB enhances SufC ATPase activity. The ATPase activity of the MTH SufB_2_C_2_ complex increased with temperature and was ~7-fold higher than that of SufC alone at 60°C. (iii) Fe-S cluster presence does not affect ATPase activity. No significant difference in ATPase activity was observed between the apo- and holo-form of either MTH SufC or the SufB_2_C_2_ complex.

Although Fe-S clusters are not detectable on SufB, their presence enhances Fe-S cluster transfer activity. Specifically, the MTH SufB_2_C_2_ complex activated apo-AcnB at about twice the rate of MTH SufC alone (Fig. 3). This enhancement was also observed with the MTH SufB_2_^C145/175/318S, H346S^C_2_ mutant complex (Fig. 3), indicating that the effect is independent of conserved cysteines in SufB. These results suggest that SufB may induce conformational changes in the SufC dimer, thereby enhancing both its ATPase activity and its ability to transfer Fe-S clusters.

## DISCUSSION

Fe-S clusters are essential cofactors involved in diverse cellular processes, including electron transport, enzyme catalysis, and gene regulation. In this study, we characterized the SufB₂C₂ complex from methanogenic archaea and demonstrated its role as a functional scaffold for Fe-S cluster assembly and transfer (42). Our findings show that the SufB and SufC from *Methanococcus maripaludis* and *Methanothermococcus thermolithotrophicus* form a stable heterotetrameric complex, SufB₂C₂, both *in vivo* and *in vitro*. This complex binds a [4Fe-4S] cluster, as evidenced by UV-visible spectroscopy, EPR analysis, and iron quantification. Importantly, the cluster is labile and transferable, and the complex can activate the apo-form of *E. coli* aconitase B (AcnB), confirming its functional role in Fe-S cluster delivery.

The interaction between the SufB₂C₂ complex and MmpX, a radical SAM enzyme involved in methanogenesis, suggests a physiological role in the maturation of Fe-S cluster-dependent enzymes. This supports the idea that the archaeal SUF system is functionally integrated into core metabolic pathways unique to methanogens and may directly transfer Fe-S clusters to target proteins.

A distinctive feature of methanogen SufC is the presence of a conserved C-terminal CX_n_CXXC motif, which is largely absent in bacterial and non-methanogenic archaeal homologs. Through mutational analysis, we identified that the three cysteines in this motif are essential for Fe–S cluster coordination and transfer (42). This result is consistent with a recent 2025 publication on *Methanocaldococcus jannaschii* SMS, which demonstrated through cryo-EM analysis that SmsC, sharing 32% sequence identity with *E. coli* SufC, binds a [4Fe-4S] cluster via three C-terminal cysteine residues that are absent in *E. coli* SufC (29). We further showed that mutations in conserved cysteine and histidine residues of SufB had no significant effect on cluster binding and transfer. These results suggest that, unlike in the *E. coli* SUF system, where both SufB and SufD contribute to cluster coordination, methanogen SufC alone serves as the primary cluster-binding component. This highlights a unique feature of the SUF system in methanogenic archaea.

Interestingly, although SufB does not bind Fe-S clusters directly, its presence significantly enhances both the ATPase activity and the cluster transfer efficiency of SufC. This enhancement is independent of SufB’s conserved cysteine residues, suggesting a structural or allosteric role. We propose that SufB binding induces conformational changes in the SufC dimer, increasing the accessibility of the Fe–S cluster and facilitating its transfer to target proteins. This is consistent with previous studies in bacterial systems showing that SufB and SufD modulate SufC activity through complex formation (43, 44).

**Fig 8:**
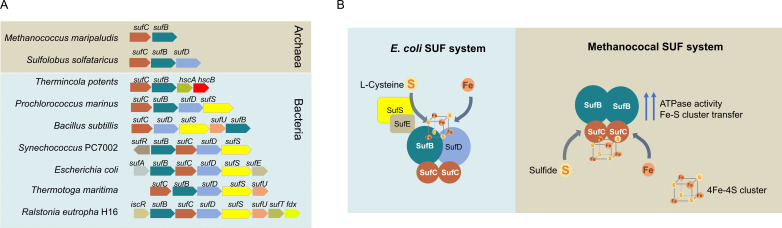
Operon structures and functional comparison of SUF systems in archaea and bacteria. (**A**) Diverse SUF operon structures in representative archaea and bacteria. Homologous genes are color-coded across species to highlight conserved components. (**B**) Proposed models of the SUF system in *E. coli* and methanococci. In *E. coli*, the SufS-SufE complex mobilizes sulfur from L-cysteine and delivers it to the SufBC_2_D scaffold for Fe-S cluster assembly. In methanococci, sulfide and Fe are assembled onto SufC within the SufB_2_C_2_ complex. SufB enhances SufC’s ATPase activity and facilitates Fe-S cluster transfer to target proteins.

From an evolutionary perspective, our findings suggest that the minimal SUF system in methanogenic archaea is active in its native host and represents an ancestral form of Fe-S cluster biogenesis (Fig. 8). Phylogenetic analyses suggest that the *sufB* and *sufC*-like genes were present in the LUCAs and have been retained in many extant archaeal lineages (26–28). The bacterial SUF system, which is phylogenetically distinct from its archaeal counterpart (26), typically includes additional proteins (e.g., SufABCDSE in *E. coli* or SufBCDSTU in *Bacillus subtilis*) that coordinate sulfur mobilization, cluster assembly, and delivery. In contrast, obligately anaerobic methanogens possess only two SUF components—SufB and SufC—yet retain full functionality in Fe-S cluster assembly and transfer (Fig. 8A). Our previous study suggested that *Methanococcus* uses sulfide instead of cysteine and cysteine desulfurase as the sulfur source for Fe-S cluster biosynthesis (22). Our findings here show the ability of SufC to independently coordinate and transfer Fe-S clusters, with SufB acting as a regulatory partner, further distinguishing the methanogen SUF system from those in bacteria (Fig. 8B). Collectively, these observations support the hypothesis that the methanococcal two-protein SUF system represents an ancient form of Fe-S cluster biogenesis that predates the Great Oxidation Event (~2.4 billion years ago), when ferrous iron and sulfide were abundant and oxidative stress was minimal (21). The later expansion of the SUF system likely evolved in response to increased O_2_ levels, necessitating more complex and protective mechanisms for Fe-S cluster assembly, for example, SufS-E for sulfur transfer and SufD for Fe acquisition (23). Thus, the methanococcal SufB₂C₂ complex not only provides mechanistic insights into Fe-S cluster biogenesis but also offers a window into the evolutionary history of this essential cellular process.

## SUPPLEMENTAL MATERIAL

Figures S1 and S2; Tables S1 to S3 spectrum.02134-25-s0001.docx Mass spectrometry, sequence alignment, conserved residues and motifs, protein constructs.

ASM does not own the copyrights to Supplemental Material that may be linked to, or accessed through, an article. The authors have granted ASM a non-exclusive, world-wide license to publish the Supplemental Material files. Please contact the corresponding author directly for reuse.
