# Supplementary material for: A minimal SufB2C2 complex functions as a [4Fe-4S] cluster scaffold in methanogenic archaea
Source: Microbiol Spectr. 2025 Oct 8;13(11):e02134-25. doi: 10.1128/spectrum.02134-25 (PMC12584684; doi:10.1128/spectrum.02134-25)
Supplement: Figures S1 and S2; Tables S1 to S3 — Mass spectrometry, sequence alignment, conserved residues and motifs, protein constructs. [file spectrum.02134-25-s0001.docx]

Figure S1


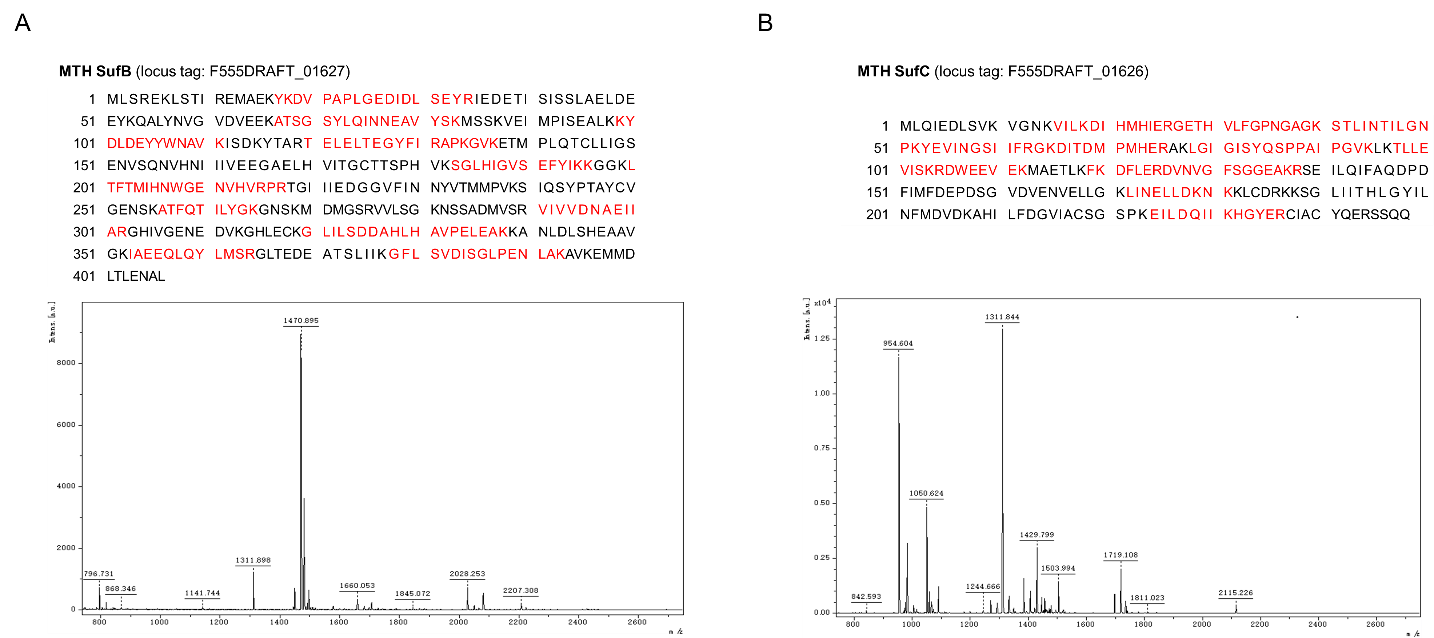


**Figure S1. Mass spectrometry analysis of proteins purified by pull-down assays in *E. coli*. (A)** Untagged MTH SufB co-purified with His_6_-tagged MTH SufC was identified by LC-MS following in-gel trypsin digestion. Matched peptides are highlighted in red, covering 41% of the SufB sequence. **(B)** Untagged MTH SufC co-purified with His_6_-tagged MTH SufB was identified by LC-MS following in-gel trypsin digestion. Matched peptides are highlighted in red, covering 55% of the SufC sequence.

Figure S2

(A)


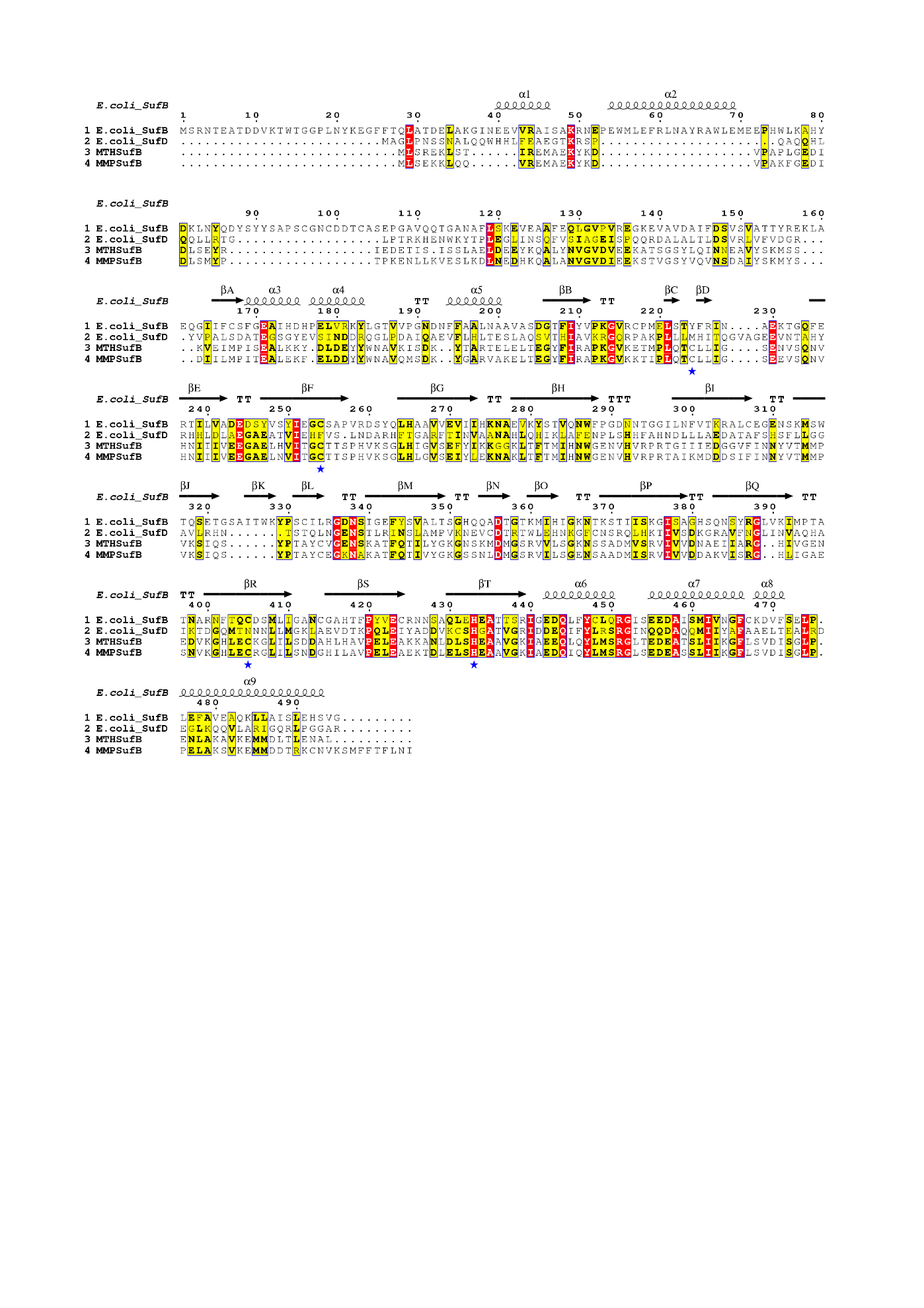


**(B)**


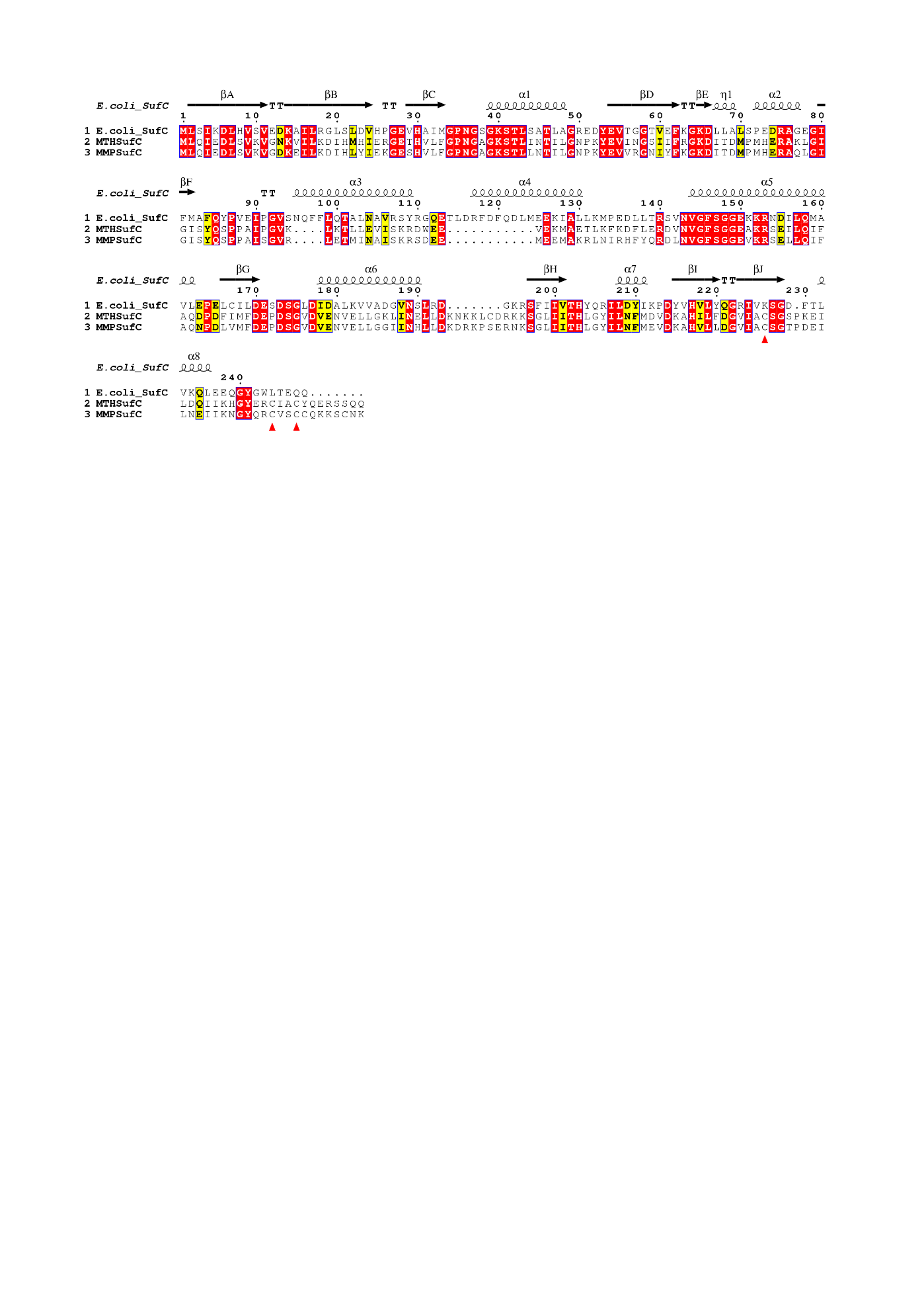


Figure S2. Sequence comparison of SufB and SufC. Identical and similar residues are highlighted in red and yellow, respectively. The secondary structures of *E. coli* proteins, based on crystal structures, are shown above the sequences using spirals (α-helices) and arrows (β-strands). (A) Sequence alignment of *E. coli* SufB/D, *Methanothermococcus thermolithotrophicus* SufB (MTH SufB), and *Methanococcus maripaludis* SufB (MMP SufB). Secondary structure elements are based on the *E. coli* SufB crystal structure (PDB ID: 5WAF). Residues potentially involved in Fe-S cluster binding are marked with blue asterisks. (B) Sequence alignment of *E. coli* SufC, *M. thermolithotrophicus* SufC (MTH SufC), and *M. maripaludis* SufC (MMP SufC). Secondary structure elements are based on the *E. coli* SufC crystal structure (PDB ID: 2D3W). The SufC residues involved in Fe-S binding, as characterized in this study, are indicated by red triangles.

**Table S1. Conserved residues in archaeal SufB homologs**

| Archaeal lineages | No. of genomes analyzed | Genomes encoding MTH SufB homologs | Homologs with C145 | Homologs with C175 | Homologs with C318 | Homologs with H346/E347 |
| --- | --- | --- | --- | --- | --- | --- |
| *Methanobacteriales* | 21 | 21 | 21 | 21 | 21 | 21 |
| *Methanococcales* | 15 | 16^a^ | 16 | 16 | 15 ^a^ | 15 ^a^ |
| *Methanopyrales* | 3 | 3 | 3 | 3 | 3 | 3 |
| *Methanocellales* | 3 | 3 | 3 | 3 | 3 | 3 |
| *Methanomicrobiales* | 12 | 12 | 12 | 11 | 11 | 12 |
| *Methanosarcinales* | 38 | 38 | 38 | 38 | 38 | 38 |
| *Methanomassiliicoccales* | 4 | 4^b^ | 3^c^ | 4 | 4 | 4 |
| All methanogens | 96 | 97 | 96 | 96 | 95 | 96 |
| non-methanogens | 196 | 196 | 30 | 193 | 196 | 196 |
| All archaea | 292 | 293 | 126 | 289 | 291 | 292 |

^a^*Methanotorris igneus* Kol 5 contains two MTH SufB homologs; only one copy contains all four conserved cysteine and histidine residues.

^b^*Candidatus Methanoplasma termitum* Mpt1 lacks an MTH SufB homolog, while *Candidatus Methanomethylophilus alvus* Mx1201 contains two.

^c^One MTH SufB homolog in *Candidatus Methanomethylophilus alvus* Mx1201 lack the conserved C145 residuel

**Table S2. Conserved motifs in archaeal SufC homologs**

| Archaeal lineages | No. of genomes analyzed | Genomes encoding MTH SufC homologs | Homologs with C-terminal CX_n_CXXC motif | Homologs with  Walker A | Homologs with  Walker B | Homologs with ABC signature | Homologs with Q loop |
| --- | --- | --- | --- | --- | --- | --- | --- |
| *Methanobacteriales* | 21 | 21 | 20^a^ | 18^g^ | 21 | 21 | 21 |
| *Methanococcales* | 15 | 15 | 14^b^ | 15 | 15 | 15 | 15 |
| *Methanopyrales* | 3 | 3 | 3^c^ | 3 | 3 | 3 | 3 |
| *Methanocellales* | 3 | 3 | 3 | 3 | 3 | 3 | 3 |
| *Methanomicrobiales* | 12 | 12 | 11^d^ | 12 | 12 | 12 | 12 |
| *Methanosarcinales* | 38 | 38 | 38 | 38 | 38 | 38 | 38 |
| *Methanomassiliicoccales* | 4 | 4 | 1^e^ | 4 | 4 | 4 | 4 |
| All methanogens | 96 | 96 | 90 | 93 | 96 | 96 | 96 |
| non-methanogens | 196 | 196 | 9^f^ | 196 | 195^h^ | 194^i^ | 196 |
| All archaea | 292 | 96 | 99 | 289 | 291 | 290 | 292 |

^a^The MTH SufC homolog in *Methanosphaera stadtmanae* DSM 3091 lacks the CX_n_CXXC motif.

^b^Five contain a CX_20_CXXC motif (*Methanocaldococcus jannaschii* DSM 2661, *Methanocaldococcus* *fervens* AG86, *Methanocaldococcus* *vulcanius* M7, *Methanocaldococcus* sp. FS406-22, and *Methanocaldococcus* *infernus* ME). The rest contain a CX_18_CXXC motif.

^c^Homologs in *Methanopyrus* *kandleri* AV19, *Methanopyrus* *sp*. SNP6, and *Methanopyrus* *sp*. KOL6 contain a CXXCX_5_C motif.

^d^The homolog in *Candidatus Methanosphaerula palustris* E1-9c lack the CX_n_CXXC motif.

^e^Only the MTH SufC homolog in *Candidatus Methanomassiliicoccus intestinalis* Issoire-Mx1 contains a CX_18_CXXC motif.

^f^The homologs in *Candidatus Korarchaeum cryptofilum* OPF8, *Archaeoglobus* *fulgidus* DSM 4304, *Archaeoglobus* *profundus* DSM 5631, *Archaeoglobus* *veneficus* SNP6, *Archaeoglobus* *sulfaticallidus* PM70-1, *Archaeoglobus* *fulgidus* DSM 8774, *Ferroglobus* *placidus* DSM 10642, *Geoglobus* *acetivorans* SBH6, and *Geoglobus* *ahangari* 234 contain a CX_18_CXXXC motif.

^g^MTH SufC homologs in *Methanothermobacter* *wolfeii* SIV6, *Methanothermobacter* *marburgensis* *str.*, and *Methanothermobacter* *thermautotrophicus* CaT2 lack the Walker A motif.

^h^The MTH SufC homolog in *Salinigranum* *rubrum* GX10 lacks the Walker B motif.

^i^ MTH SufC homologs in *Sulfolobus solfataricus P2* and *Salinigranum* *rubrum* GX10 lack the ABC signature.

**Table S3. Protein expression constructs**

| **Protein constructs** | **Host** | **Plasmids** |
| --- | --- | --- |
| His-tagged MTH SufC | *E. coli* Rosetta 2(DE3) | pET15b-NHis-MTHSufC |
| His-tagged MTH SufC (C218S) | *E. coli* Rosetta 2(DE3) | pET15b-NHis-MTHSufC(C218S) |
| His-tagged MTH SufC (C237S) | *E. coli* Rosetta 2(DE3) | pET15b-NHis-MTHSufC(C237S) |
| His-tagged MTH SufC (C240S) | *E. coli* Rosetta 2(DE3) | pET15b-NHis-MTHSufC(C240S) |
| His-tagged MTH SufB | *E. coli* Rosetta 2(DE3) | pDCH-CHis-MTHSufB |
| His-tagged MTH SufB_2_C_2_ | *E. coli* Rosetta 2(DE3) | pET15b-NHis-MTHSufC+pDCH-MTHSufB |
| His-tagged MTH SufCB | *E. coli* Rosetta 2(DE3) | pET15b-MTHSufC+pDCH-CHis-MTHSufB |
| His-tagged MTH SufB_2_^C145/175/318S,H346S^C_2_ | *E. coli* Rosetta 2(DE3) | pET15b-NHis-MTHSufC+pDCH-MTHSufB(C145/175/318SH346S) |
| His-tagged MTH SufB_2_C_2_^C218/237/240S^ | *E. coli* Rosetta 2(DE3) | pET15b-NHis-MTHSufC(C218/237/240S)+pDCH-MTHSufB |
| His-tagged MTH SufB_2_^C145/175/318S,H346S^C_2_^C218/237/240S^ | *E. coli* Rosetta 2(DE3) | pET15b-NHis-MTHSufC(C218/237/240S)+pDCH-MTHSufB(C145/175/318SH346S) |
| Strep-tagged MTH SufC | *E. coli* Rosetta 2(DE3) | pET15b-Nstrep-MTHSufC |
| Strep-tagged MTH SufB_2_C_2_ | *E. coli* Rosetta 2(DE3) | pET15b-Nstrep-MTHSufC+pDCH-MTHSufB |
| Strep-tagged MTH SufB_2_^C145/175/318S,H346S^C_2_ | *E. coli* Rosetta 2(DE3) | pET15b-Nstrep-MTHSufC+pDCH-MTHSufB(C145/175/318SH346S) |
| Strep-tagged MTH SufB_2_C_2_^C218/237/240S^ | *E. coli* Rosetta 2(DE3) | pET15b-Nstrep-MTHSufC(C218/237/240S)+pDCH-MTHSufB |
| Strep-tagged MTH SufB_2_^C145/175/318S,H346S^C_2_^C218/237/240S^ | *E. coli* Rosetta 2(DE3) | pET15b-Nstrep-MTHSufC(C218/237/240S)+pDCH-MTHSufB(C145/175/318SH346S) |
| Strep-tagged MTH SufB_2_C_2_^K40R^ | *E. coli* Rosetta 2(DE3) | pET15b-Nstrep-MTHSufC (K40R)+pDCH-MTHSufB |
| MMP SufB_2_C_2_ | *M. maripaludis* S01 | pMEV4mTs-Nstrep-MMPSufCB |
| MMP MmpX | *M. maripaludis* Δ*mmpX* | Pst-pMEV4mTs-cTAP-MMPX |
